# Synergy and Other Interactions between Polymethoxyflavones from Citrus Byproducts

**DOI:** 10.3390/molecules201119677

**Published:** 2015-11-06

**Authors:** Benito F. García, Ascensión Torres, Francisco A. Macías

**Affiliations:** Allelopathy Group, Department of Organic Chemistry, Institute of Biomolecules (INBIO), Campus de Excelencia Internacional (ceiA3), Faculty of Science, University of Cadiz, C/República Saharaui n 7, 11510 Puerto Real, (Cádiz), Spain; benito.fernandez@uca.es (B.F.G.); chon.torres@uca.es (A.T.)

**Keywords:** citrus by-products, flavonoids, synergism, isobolography, DPPH, coleoptiles

## Abstract

The citrus by-products released from citrus processing plants may contain high levels of potentially bioactive compounds such as flavonoids, which are a widely distributed group of polyphenolic compounds with health-related properties based on their antioxidant activity. In the study reported here, the potential bioactivities and antioxidant activities of extracts, fractions and compounds from citrus by-products were evaluated along with the chemical interactions of binary mixtures of compounds and complex mixtures. The bioactivities and interactions were evaluated in wheat coleoptile bioassays and the antioxidant activity was evaluated by the al DPPH (2,2-diphenyl-1-picrylhdrazyl radical) radical scavenging assay. The extracts, fractions and most of the isolated compounds (mainly polymethoxyflavones) showed high activity in the wheat coleoptile bioassay. However, the antioxidant activity was not consistently high, except in the acetone extract fractions. Moreover, a study of the interactions with binary mixtures of polymethoxyflavones showed the occurrence of synergistic effects. The complex mixtures of fractions composed mainly of polymethoxyflavones caused a synergistic effect when it was added to a bioactive compound such as anethole. The results reported here highlight a new application for the wheat coleoptile bioassay as a quick tool to detect potential synergistic effects in compounds or mixtures.

## 1. Introduction

The fruits of the *Citrus* species are important crops because of their industrial value, especially in foods and cosmetics. Large amounts of by-products, which are composed of peel and seeds, are produced every year in citrus processing plants and these may contain high levels of potentially bioactive compounds. Most bioactive compounds are found in the peel and the inner white pulp [1,2]. Phytochemical investigations into *Citrus* species have shown that the peel contains flavonoids, limonoids and coumarins [1,3]. Flavonoids are a widely distributed group of bioactive compounds and some of them, such as hesperidin, naringin and polymethoxyflavones (PMFs), are characteristic of citrus plants, while others, such as rutin and quercetin, are common in the plant kingdom [2]. *Citrus* flavonoids, especially PMFs, are flavones that bear two or more methoxy groups on the basic benzo-γ-pyrone (15-carbon, C_6_-C_3_-C_6_) skeleton, which has a carbonyl group at the C_4_ position. PMFs are major constituents of citrus peel [4] and they have attracted considerable attention because they have also exhibited a wide spectrum of biological activity, including anticancer [5,6], anti-inflammatory [7,8], antioxidant [9,10], antimutagenic [11,12] and antimicrobial [13,14] activities. Most studies into the biological activities of PMFs have focused on nobiletin [15,16], tangeretin [17,18] and sinensetin [19,20].

The longstanding, successful use of herbal drug combinations in traditional medicine demands that a rationale be found for their comparative pharmacological and therapeutic superiority to isolated single constituents. The assessment of synergy has become a key area in phytomedicine research in recent years.

The effects of such preparations can result from the interaction of an array of chemically diverse components, the identities and concentrations of which may vary from one preparation to the next. Proponents of the medicinal use of botanicals often argue that their components interact synergistically, such that the combined effect is greater than the sum of the parts. Indeed, there are many examples in the literature in which constituents are more effective than the isolated compound in the phytochemical matrix [21].

Chemotherapy has also seen a gradual transition from the long and passionately advocated mono-substance therapy toward multidrug therapy. It is becoming increasingly clear through observation that many diseases possess a multi-causal etiology and a complex pathophysiology, which can be treated more effectively with well-chosen drug combinations than with a single drug. Multidrug therapy is now practiced worldwide in the treatment of AIDS and other infectious diseases, hypertension, numerous types of cancer and rheumatic diseases [22].

Although very different activities have been reported for flavonoids in numerous publications, it is now believed that these compounds may have a role in increasing the biological activity of other compounds by synergistic or other mechanisms [23]. Pairs of flavonoids, such as genistein, baicalein, hesperetin, naringenin and quercetin, have proven to have synergistic effects on the inhibition of the growth of human breast cancer cells [24]. In a study on the effect of PMFs (6,7,4′,5′-tetramethoxy-5-monohydroxyflavone, 5,6,8,3′,6′-pentamethoxyflavone, 5,6,7,3′,4′,5′-hexamethoxyflavone) on the degranulation in RBL-2H3 cells, all of these PMFs suppressed the degranulation from Ag-stimulated RBL-2H3 but combined PMF treatments enhanced the inhibition of degranulation compared to treatments with single PMFs [25]. Likewise, nobiletin had a preventive effect on H_2_O_2_-induced apoptosis in human neuroblastoma SH-SY5Y cells and a binary mixture of tangeretin and 5-demethylnobiletin showed an inhibitory effect on growth. These three PMFs in different combinations as binary mixtures also exhibited synergistic effects [26].

In other studies on PMF-rich fractions obtained from the peel of *Citrus sunki*, greater antiproliferative activity in HL-60 cells was found in comparison to that observed for any single PMF [27]. Synergistic effects have recently been described for the anti-inflammatory activity on RAW 264.7 cells (tumor cell line) with a binary mixture of nobiletin and sulforaphane or with combinations of other flavonoids [28,29]. In studies of drug interactions in chemotherapy it has been found that combinations of nobiletin with the drugs paclitaxel and carboplatin generate a synergetic effect in inhibiting the proliferation of carcinoma cell lines A549 and human H460 [30].

Peels and seeds, both of which are by-products of the juice extraction industry, are interesting sources of phenolic compounds that include phenolic acids and flavonoids [31]. These compounds exhibit antioxidant activity in different ways: e.g., antiradical (^•^OH, O_2_^•^^−^), antilipoperoxidation (R^•^, ROO^•^, RO^•^), antioxygen (O_2_, ^1^O_2_) and metal chelating activities [32]. Flavonoids are potential antioxidants against free radicals as they act as radical scavengers. The methanolic extract of citrus peel has been shown to possess strong antioxidant activity in assays with the 2,2-diphenyl-1-picrylhaydrazyl (DPPH) radical, hydroxyl radical and intracellular reactive oxygen species (ROS), along with scavenging and DNA damage inhibition [33]. A similar study was carried out on the antioxidant activity of a methanolic extract of by-products from citrus that was prepared using a novel drying technique known as high speed drying (HSD). The extract contained large amounts of polymethoxylated flavones (heptamethoxyflavone and nobiletin) and flavones (hesperidin and naritutin) and it showed strong activities on radical scavenging and lipid peroxidation inhibition [34].

One of the objectives proposed in this paper is the application of the wheat coleoptile bioassay as a quick tool to detect possible synergistic effects in compounds or mixtures thereof. This bioassay has the advantage of being a rapid test (24 h) that is sensitive to a wide range of bioactive substances, including plant growth regulators, herbicides, antimicrobials, mycotoxins and assorted pharmaceuticals [35,36,37].

The study reported here concerns a bioassay-guided isolation procedure to characterize the main bioactive compounds present in extracts obtained from citrus waste from *Citrus sinensis*. Furthermore, the chemical interactions of binary mixtures of the major compounds were investigated along with the influence on the bioactivity in the wheat coleoptile bioassay of a bioactive complex mixture formed by adding an active compound. The antioxidant capacity of dichloromethane and acetone extracts, the most active fractions, and the isolated polymethoxyflavones were also analyzed.

## 2. Results and Discussion

### 2.1. Isolation of Compounds

The raw material was the residue from the production of citrus juice from *Citrus sinensis*, variety Valencia Late, supplied by the Company Lara (Laranjo Do Algarbe-LDA, Silves, Portugal), which is located in Southern Portugal. The fruit was obtained during the 2003 and 2004 campaigns. The techniques used to obtain the extracts from citrus waste were steam distillation and maceration in dichloromethane and acetone on a semi-industrial scale at the EVESA S.A. installations (La Línea de la Concepción, Cádiz, Spain). Raw samples were defatted with hexane prior to maceration in dichloromethane and acetone. The extracts were subsequently fractionated by column chromatography on silica gel using as eluent hexane/ethyl acetate (0%–100%) and 100% acetone in increasing polarity. The resulting fractions were concentrated, with cooling in an ice bath in the case of the essential oils extract. The essential oils extract (10 g) provided four fractions: A (volatile compounds, 8.9 g), B (611 mg), C (340 mg) and D (54 mg). The dichloromethane extract (15 g) provided nine fractions: A′ (1 g), B′ (4.3 g), C′ (697 mg), D′ (462 mg), E′ (507 mg), F′ (805 mg), G′ (1.4 g), H′ (1.3 g) and I′ (1.2 g). The acetone extract (5 g) provided five fractions: A′′ (1.7 g), B′′ (2.1 g), C′′ (93 mg), D′′ (620 mg) and E′′ (448 mg).

**Figure 1:**
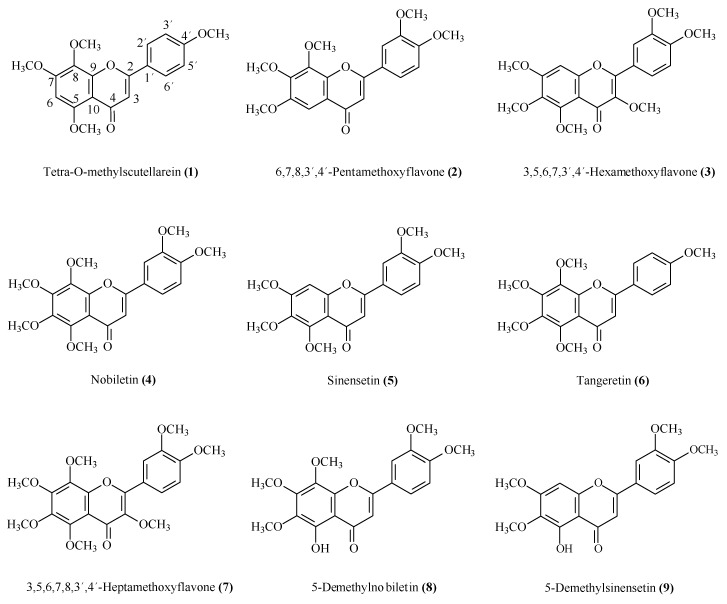
Structures of the flavonoids **1**–**9** isolated from citrus waste of *Citrus sinensis*.

**Figure 2:**
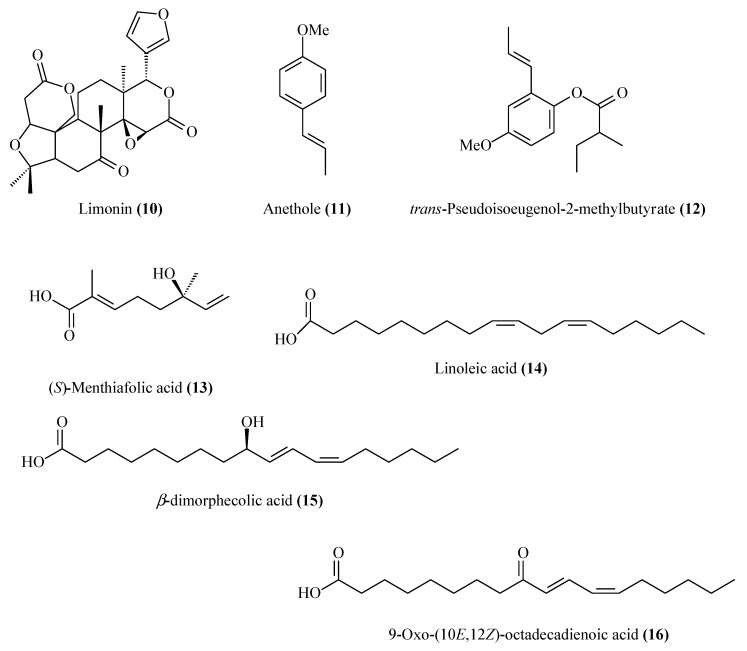
Structures of limonoid **10**, phenylpropanoids **11** and **12**, and acids **13**–**16** isolated from the citrus waste of *Citrus sinensis*.

A total of 16 compounds were isolated from the most active fractions (Figure 1 and Figure 2) in successive purifications by HPLC with different eluent mixtures (hexane/acetone; hexane/EtOAc). These compounds were identified from their spectroscopic data (^1^H-NMR, ^13^C-NMR, IR, and MS) by comparison with the data reported in the literature for tetra-*O*-methylscutellarein (**1**) [38], 6,7,8,3′,4′-pentamethoxyflavone (**2**) [39], 3,5,6,7,3′,4′-hexamethoxyflavone (**3**) [38], nobiletin (**4**) [39], sinensetin (**5**) [39], tangeretin (**6**) [39], 3,5,6,7,8,3′,4′-heptamethoxyflavone (**7**) [39], 5-demethylnobiletin (**8**) [39], 5-demethylsinensetin (**9**) [40], limonin (**10**) [41], anethole (**11**) [42], *trans*-pseudoisoeugenol-2-methylbutyrate (**12**) [43], (*S*)-menthiafolic acid (**13**) [44], linoleic acid (**14**) [45], β-dimorphecolic acid (**15**) [46] and 9-oxo-(10*E*,12*Z*)-octadeca-10,12-dienoic acid (**16**) [47] (Figure 1 and Figure 2).

Several of the isolated compounds **1**–**16** have been described previously from citrus fruit. The flavonoids **1**, **2**, **3**, **4**, **5**, **6**, **7** and **8** have been identified in peel, leaves and stems of *Citrus* spp. [38,39] and [48,49]; the limonoid **10** was identified in the seeds of *Citrus* spp. [50,51]; the phenylpropanoid **11** is present in the leaves of mandarin and bergamot [52] and the fatty acid **14** in citrus fruit [53].

The remaining compounds are reported for the first time from citrus fruit although they have been identified previously in others plants. The flavonoid **9** was found in the aerial parts of *Cetaurea napifolia* L. [40], the phenylpropanoid **12** in the genus *Pimpinella* [54,55], the monoterpene acid **13** in the fruit of *Euterpe oleracea* [56], and fatty acids derivatives **15** in *Dimorphotecha aurantiaca* [57] and **16** in *Pisum sativum* [58].

### 2.2. Coleoptile Bioassay Results from Extracts, Fractions and Isolated Compounds

The essential oils, dichloromethane and acetone extracts were subjected to a bioassay-guided isolation using the etiolated wheat coleoptile bioassay to obtain active fractions and for the isolation of the main bioactive compounds from the citrus waste of *Citrus sinensis*. The bioassays were carried out at concentrations of 1.0, 0.5, 0.25, 0.125 and 0.075 mg·mL^−1^ for extracts and fractions, and at 1.0, 0.3, 0.1, 0.03 and 0.01 mM for pure compounds.

**Figure 3:**
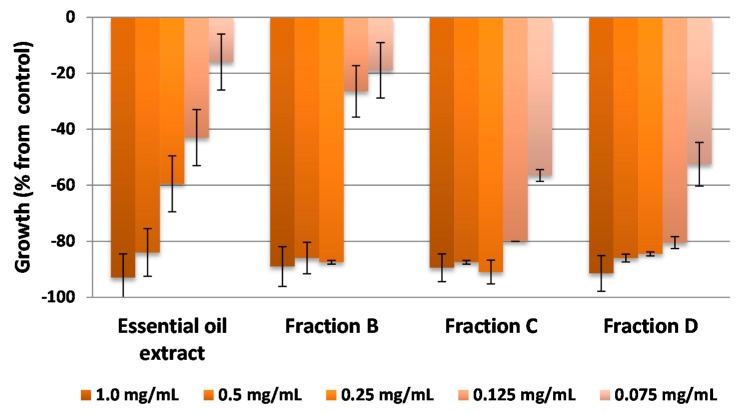
Bioactivities obtained in the etiolated wheat coleoptile bioassay for the essential oils extract and the fractions obtained by chromatography from citrus waste from *Citrus sinensis.* Values are expressed as percentage difference from control.

**Figure 4:**
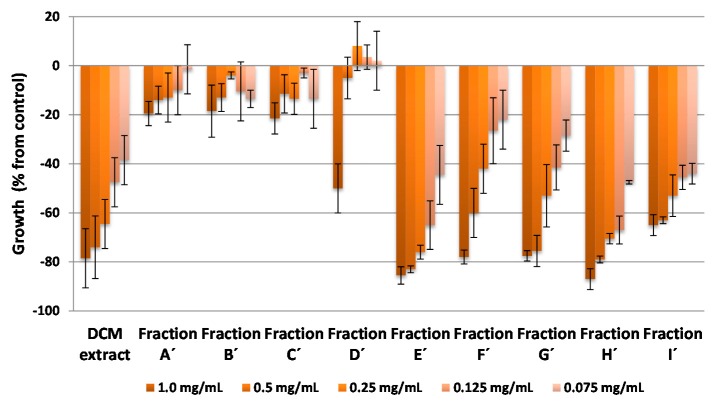
Bioactivities obtained in the etiolated wheat coleoptile bioassay for the dichloromethane extract and the fractions obtained by chromatography of the citrus waste from *Citrus sinensis.* Values are expressed as percentage difference from control.

**Figure 5:**
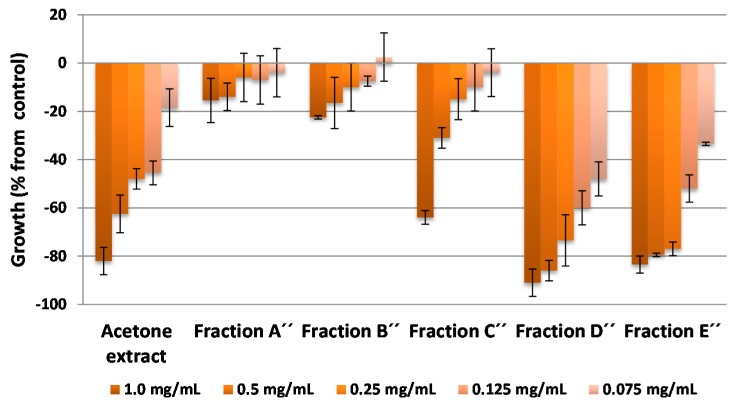
Bioactivities obtained in the etiolated wheat coleoptile bioassay for the acetone extract and the fractions obtained by chromatography from citrus waste of *Citrus sinensis.* Values are expressed as percentage difference from control.

**Figure 6:**
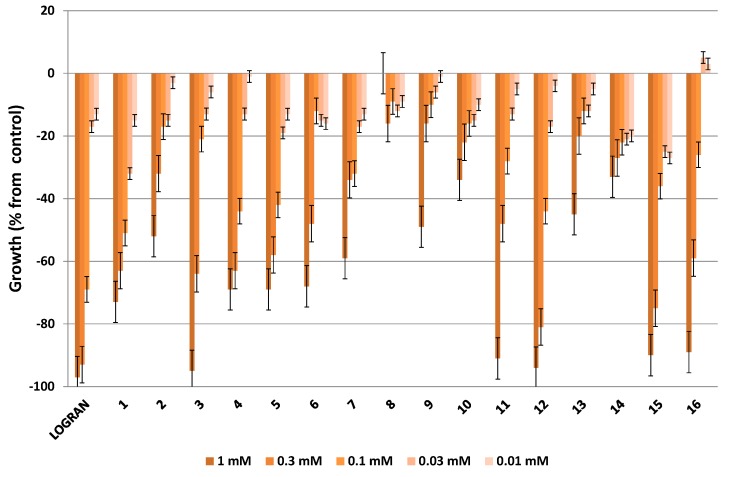
Effect of compounds **1**–**16** from the bioactive fractions of the different extracts of *Citrus sinensis* on the elongation of etiolated wheat coleoptiles. Values are expressed as percentage difference from control.

The etiolated wheat coleoptile bioassay was used as an initial approach to evaluate the bioactivity of the extracts, fractions and pure compounds The wheat coleoptile bioassay is a rapid test (24 h) that is sensitive to a wide range of bioactive substances, including plant growth regulators, herbicides, antimicrobials, mycotoxins and assorted pharmaceuticals [35,36,37]. The results of these bioassays are represented in Figure 3, Figure 4, Figure 5 and Figure 6, where negative values signify inhibition, positive values denote activation and zero represents the control.

The essential oils extract showed the highest inhibition, with a value of 93.0% obtained at 1.0 mg·mL^−1^, and its activity profile decreased uniformly with dilution to give a reasonably high value (60% inhibition) at 0.25 mg·mL^−1^ (Figure 3). Fractions B, C and D generally showed better inhibition activity profiles than the initial extract as the activity was retained upon dilution. The three fractions exceeded 80% inhibition at the first three dilutions (1.0, 0.5, 0.25 mg·mL^−1^) and for fractions C and D this activity was even maintained at the fourth dilution (0.125 mg·mL^−1^).

Purification of fractions B and C gave two major phenylpropanoids **11** (from B, 179.2 mg) and **12** (from C, 98 mg). Purification of fraction D gave the following polymethoxyflavones: **1** (13.9 mg), **3** (1.9 mg), **4** (18 mg), **5** (5.4 mg), **6** (9.5 mg) and **7** (1.8 mg).

The activity profiles of the dichloromethane (DCM) extract and the fractions obtained from it (A′–I′) are shown in Figure 4. The DCM extract showed a high inhibitory activity, close to 80% at 1.0 mg·mL^−1^, and a good activity profile with dilution—even better than that of the essential oils extract. A value close to 40% inhibition was obtained at 0.075 mg·mL^−1^. The activity of this extract is reflected in the activities shown by fractions E′, F′, G′, H′ and I′ (the inhibition values for fractions E′ to H′ at 1.0 mg·mL^−1^ were around 80%). Fractions E′ and Hʹ showed higher activity levels, *i.e.*, above 80% inhibition at the highest concentration (1.0 mg·mL^−1^), and this high activity was retained at lower concentrations (around 60% for the fourth dilution, 0.125 mg·mL^−1^).

The purified compounds from the most active fractions (E′–I′) and their distributions are shown in Table 1. It is noteworthy that one of the most active fractions (E′) contains only three major compounds, *i.e.*, polymethoxyflavone **8** and fatty acids **15** and **16**, while the next most active fraction (H′) contains appreciable amounts of seven polymethoxyflavones (**1**, **3**, **4**, **5**, **6**, **7**, **9**) and limonin (**10**).

**Table 1:** Amounts of compounds isolated from the most active fractions of the dichloromethane extract.

Fraction	Compounds (mg)															
	1	2	3	4	5	6	7	8	9	10	13	15	16
**E′**	-	-	-	-	-	-	-	2.0	-	-	-	12.4	9.2
**F′**	-	-	-	-	-	7.9	9.6	-	2.8	14.1	4.8	-	-
**G′**	56.1	-	10.8	49.2	-	33.1	12.3	1.4	6.4	43	-	-	-
**H′**	43.6	-	31.8	309.9	111.6	3.6	10.5	-	4.9	2.4	-	-	-
**I′**	3.1	80.3	5.1	22.7	33.4	1.0	1.4	1.2	1.4	-	-	-	-

The acetone extract showed a high inhibitory activity on coleoptile elongation at 1.0 mg·mL^−1^ (>80%) but, in comparison to the activity profile of the DCM extract, the activity of the acetone extract decreased more markedly with dilution (Figure 5). However, the active fractions (D′′ and E′′) showed better activity profiles than the initial extract. For example, the inhibition levels for these fractions at 1.0, 0.5 and 0.25 mg·mL^−1^ were 91.0, 86.0 and 73.5%, respectively, for fraction D′′ and 83.5%, 79.5% and 77.0%, respectively, for fraction E′′.

The amounts of purified compounds obtained from the most active fractions D′′ and E′′ are shown in Table 2. In this case there is little variability in the compositions of the products in the two fractions and the only difference concerns the relative amounts in each fraction, with the prevalence of polymethoxyflavones **1**–**9**.

**Table 2:** Amounts of compounds isolated from the most active fractions of the acetone extract.

Fraction	Compound (mg)								
	1	3	4	5	6	7	8	9	14
**D′′**	1.9	-	2.4	-	1.6	11.8	1.8	1.9	5.0
**E′′**	6.8	3.9	70.1	16.6	2.6	3.6	-	-	-

The effects of compounds **1**–**16**, which were isolated from the bioactive fractions of the different extracts of *Citrus sinensis*, on the elongation of etiolated wheat coleoptiles at dilutions in the range 1.0 to 0.01 mM are represented in Figure 6. The commercial herbicide Logran was used as an internal reference [59]. All of the compounds showed inhibitory activity and more than half of them gave activity results that are of interest.

Of all the isolated compounds, polymethoxyflavone **3**, two phenylpropanoids (**11** and **12**) and two fatty acids (**15** and **16**) showed the highest inhibitory activity on coleoptile elongation, with almost 90% activity at 1 mM. However, this activity decreased significantly with dilution for all of these compounds with the exception of compound **12**, which was even active at the second concentration tested (at 0.3 mM with 81.0%). The activity values for these compounds at 1 mM were similar to that of the commercial herbicide Logran. The phenylpropanoids **11** and **12** have proven to have diverse biological activities, such as antioxidant, bactericidal, fungicidal and anti-inflammatory activities [60,61]. The hydroxydienoic (**15**) and cetodienoic (**16**) acids are the most common by-products of lipid peroxidation derivatives from linoleic acid. These acids play an important role in biological systems and have been isolated from both plants and animals. Several studies have been carried out on the biological activities of compounds **15** and **16**, including the study of quimiotax, hyperproliferative effects, regulation of phospholipase activity, regulation of cell adhesion, and their incorporation into phospholipids and thus their involvement in the biological regulation membranes [62].

Five polymethoxyflavones (**1**, **4**, **5**, **6** and **7**) can be placed in a second group that showed around 60% inhibitions or higher at the same concentration. In the introduction it was stated that polymethoxyflavones are the major constituents of citrus peel and these compounds have attracted considerable attention because of the wide spectrum of their biological activities, which include anticancer, anti-inflammatory, antioxidant, antimutagenic and antimicrobial activities [5,6,7,8,9,10,11,12,13,14,15,16,17,18,19,20].

The most active compounds isolated from citrus wastes belong to different chemical classes and these were mainly six polymethoxyflavones (compounds **1**, **3**–**7**), two phenylpropanoids **11**, **12** and two fatty acids **15**, **16**. It is clear that the inhibitory activity exhibited by these isolated compounds compared to that shown by the fractions and extracts from which they were obtained is not a simple phenomenon to explain. In some cases it can be assumed that a major single compound, such as phenylpropanoids **11** and **12** (179.2 mg and 98 mg from fractions B and C, respectively) from the essential oil extract, is responsible for the observed behavior, so it is also possible that a small number of such compounds could exert the effect. The activity of compounds found in the citrus waste fractions makes it conceivable that they are involved in some types of interaction. Activity caused by synergistic or additive interactions is usually not comparable to the activity of a single active compound, unless such a compound already participates in the combination. The specific structural factors that determine the activity of a particular combination of compounds remains unclear. The same holds true for the combined effect because the nature of such an effect cannot be predicted on the basis of an individual compound acting in isolation. In some cases, a non-inhibitory concentration of a specific compound inhibits growth when this compound acts additively or synergistically with other compounds that are present and such joint action is the most common situation. One of the aims of the study presented here was to clarify these interaction effects by carrying out etiolated wheat coleoptile bioassays on binary mixtures of compounds and fractions.

### 2.3. DPPH Radical Scavenging Assay with Extracts, Fractions and Compounds

Antioxidant compounds in samples react with the DPPH radical, which is a nitrogen-centered radical, and convert it to 1,1-diphenyl-2-picryl hydrazine at a very rapid rate due to its hydrogen-donating ability. The 2,2-diphenyl-1-picrylhydrazyl radical (DPPH·) is a commercially available stable organic nitrogen radical that has a deep-purple color. The radical scavenging activity (RSA) assay measures the reducing capacity of antioxidants towards DPPH·. Upon reduction, the color of the DPPH· solution fades and this color change is conveniently monitored spectrophotometrically at 517 nm. When a solution of DPPH is mixed with a substance that can donate a hydrogen atom, the reduced form of the radical is generated and this change is accompanied by loss of color. The antioxidant activity index (AAI) allows the antioxidant capacity of extracts and pure compounds to be compared regardless of the concentration of DPPH· and the solvent used by setting ranges of values that can classify the antioxidant activity of the sample.

**Figure 7:**
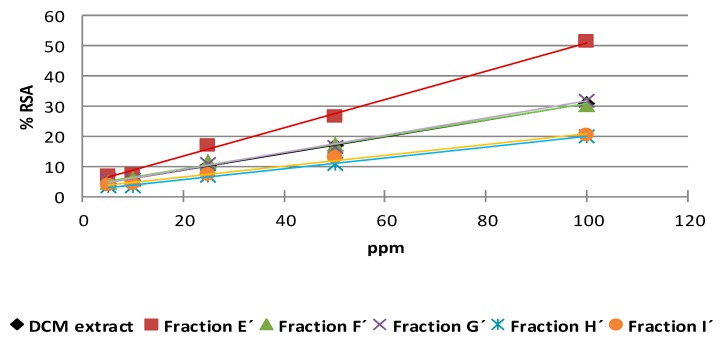
DPPH radical scavenging activity of the dichloromethane extract and its fractions from citrus by-products: Fraction E′ showed the highest scavenging activity; Fractions F′ and G′ showed the same antioxidant activity as the DCM extract; Fractions H′ and I′ showed the lowest scavenging activity.

**Figure 8:**
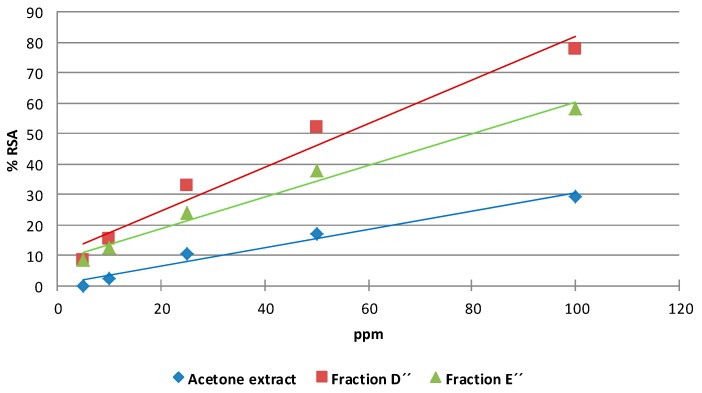
DPPH radical scavenging activity of the acetone extract and its fractions from citrus by-products. Fraction D′′ showed the highest scavenging activity. Fraction E′′ showed medium activity antioxidant. The acetone extract showed the lowest scavenging activity.

The DPPH radical scavenging potentials of different concentrations of acetone and dichloromethane extracts and the different bioactive fractions obtained from them are depicted in Figure 7 and Figure 8. The samples showed a dose-dependent activity on DPPH radical scavenging. The antioxidant activity of a sample is attributed to its hydrogen-donating ability.

The results show that the extracts and different fractions exhibited dose-dependent antioxidant activity. The fractions from the acetone extract showed % RSA values higher than 50% at 100 ppm. The antioxidant activity index (AAI) values for the fractions from the acetone extract classify them as having strong antioxidant activity (Table 3).

**Table 3:** Antioxidant activity index (AAI) values for fractions of the acetone extract from citrus by-products.

Fraction	IC_50_ (µg/mL)	AAI	Antioxidant Activity
Fraction D′′	58.01	1.43 (±0.04)	Strong
Fraction E′′	78.31	1.10 (±0.06)	Strong

These results are consistent with those published previously [63], where the differences observed between fractions and the original extract reflect the variation of the phytochemical composition of the samples, a trend that is mentioned in various publications on citrus antioxidants [64,65]. The changes in the concentrations of the compounds and their proportions in fractions can lead to the appearance of synergistic effects, which have been observed in several cases [66,67].

**Figure 9:**
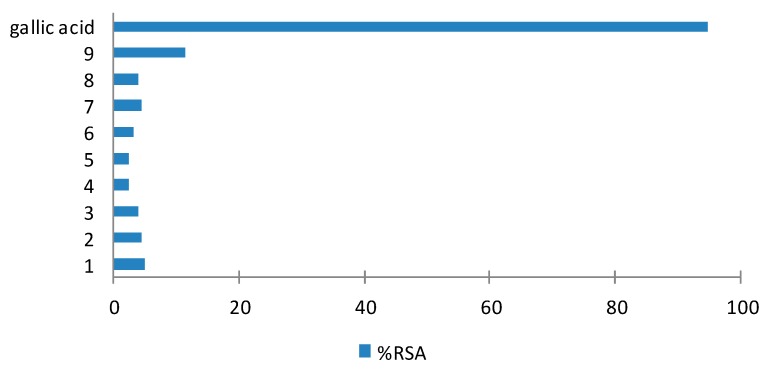
DPPH radical scavenging activity of polymethoxyflavones (**1**–**9**) at 25 µM.

The DPPH free radical scavenging potentials were tested for the major compounds from the different fractions, namely the polymethoxyflavones **1**–**9** at a concentration of 25 µM (Figure 9). Gallic acid was used as a positive control and this showed the highest activity (94.91% RSA; AAI = 27 (very strong antioxidant activity)) [68]. The radical scavenging polymethoxyflavones showed very low activities, with 5-demehylsinenstin (**9**) showing the highest % RSA (11.44) of the series.

The data obtained are consistent with the typical radical scavenging potentials of flavonoids. It is generally accepted that the antioxidant activity of flavonoids is mainly caused by the presence of the 2,3-double bond in conjugation with a 4-oxo function, the *o-*dihydroxy structure at positions 3′,4′, and the presence of two hydroxyl groups in positions 3 and 5 [69]. The flavonoids tested here do not contain hydroxyl groups at these positions and only 8 and 9 bear a hydroxyl group at all, in these cases in position 5. Therefore, the antioxidant activities of the acetone fractions may be due to the presence of unidentified compounds or possible synergistic effects between them.

### 2.4. Isobolographic Analysis of Chemical Interaction in Coleoptile Bioassay

Studies on binary-mixture are often conducted with the aim of elucidating the effect of one specific chemical on the biological action of another. The results can be interpreted in relation to reference models by the use of isobolograms. However, the amount of data needed for these analyses is large and such experiments are therefore rarely repeated. The joint effect of the majority of chemical mixtures can be predicted using the reference model named “Independent Similar Action by Bliss” [70]. The approach becomes challenging, however, when the mixtures include chemicals that synergize or antagonize the effects of other components [71].

The study described here involved an investigation into the reproducibility of isobolograms for binary mixtures in the elongation of the etiolated wheat coleoptile in terms of deviation from the reference model, dose-level dependence, combinations of different proportions and isobole asymmetry. The data employed were obtained from different binary mixtures of compounds isolated from by-products of *Citrus sinensis* tested in the coleoptile bioassay, as well as complex mixtures made from active fractions and an active compound.

The isobole method is independent of the mechanism of action and applies under most conditions. It also makes no assumptions as to the behavior of each agent and is therefore applicable to multiple component mixtures. An isobole is an “iso-effect” curve, in which a combination of constituents (da, db) is represented on a graph, the axes of which are the dose-axes of the individual agents (Da and Db). If the agents do not interact, the isobole (the line joining the points that represent the individual doses with the same effect as the combination) will be a straight line (line of additivity). If synergy is occurring, *i.e.*, the effect of the combination is greater than expected from their individual dose-response curves, the dose of the combination needed to produce the same effect will be less than for the sum of the individual components and the point is located below the line of additivity. The opposite applies for antagonism, in which the dose of the combination is greater than expected and is located above the line of additivity.

Isobolographic analysis was performed to evaluate the interactions between the compounds [72]. The type of interaction is evaluated from the values of the interaction index (λ); values close to **1** correspond to an additive interaction, values higher than **1** imply an antagonistic interaction, and values less than 1 indicate a synergistic interaction. The difference between the interaction index and a value of one represents the intensity or magnitude of the synergistic or antagonistic effect.

After examining interactions of 44 binary mixtures with the isolated compounds from *Citrus* *sinensis* on the etiolated wheat coleoptile bioassay, and applying Student’s *t* test with a confidence level of 90%, 32 additive interactions, 7 synergistic interactions and 5 antagonistic interactions were detected (see Table 4).

**Table 4:** Types of interactions with Student’s *t* test (α = 0.1) and interaction index (λ) between the different combinations of binary mixtures of isolated compounds from *Citrus sinensis* in the etiolated wheat coleoptile bioassay (Add.: additive; Syn.: synergistic; Ant.: antagonistic).

Binary Mixture of Compound	Combination Ratio																	
	9:1		4:1		3:1		2:1		1:1		1:2		1:3		1:4		1:9	
	*ED25*	λ	*ED25*	λ	*ED25*	λ	*ED25*	λ	*ED25*	λ	*ED25*	λ	*ED25*	λ	*ED25*	λ	*ED25*	λ
4:5	Add.	1.6	-	-	Add.	1.1	-	-	Syn.	0.3	-	-	Syn.	0.2	-	-	Syn.	0.4
4:2	-	-	-	-	-	-	-	-	Add.	1.2	Ant.	2.3	-	-	Ant.	5.3	Ant.	1.9
4:1	Add.	0.7	Add.	0.6	-	-	Add.	0.6	-	-	Add.	1.1	-	-	-	-	-	-
1:2	-	-	-	-	Add	1.2	-	-	Syn.	0.5	-	-	-	-	-	-	Ant.	1.8
1:5	-	-	-	-	Add.	1.6	-	-	Add.	2.9	-	-	-	-	-	-	-	-
5:10	-	-	-	-	Syn.	0.0	-	-	Syn.	0.2	-	-	Syn.	0.1	-	-	-	-
4:10	-	-	-	-	Add.	1	-	-	Add.	0.8	-	-	Add.	0.7	-	-	-	-
1:10	-	-	-	-	Add.	1	-	-	Add.	1.2	-	-	Add	1.6	-	-	-	-
	*ED50*	λ	*ED50*	λ	*ED50*	λ	*ED50*	λ	*ED50*	λ	*ED50*	λ	*ED50*	λ	*ED50*	λ	*ED50*	λ
11:6	-	-	-	-	Add.	1.1	-	-	Add.	0.9	-	-	Ant.	2.9	-	-	-	-
11:1	-	-	-	-	Add.	0.8	-	-	Add.	0.8	-	-	Add.	0.6	-	-	-	-
11:4	-	-	-	-	Add.	1.1	-	-	Add.	0.7	-	-	Add.	0.7	-	-	-	-
11:7	-	-	-	-	Add.	2.4	-	-	Add.	1.5	-	-	-	-	-	-	-	-
11:9	-	-	-	-	Add.	0.9	-	-	Add.	0.9	-	-	Add.	1.4	-	-	-	-
11:16	-	-	-	-	Add.	1.1	-	-	Add.	0.9	-	-	Add.	1.2	-	-	-	-

**Figure 10:**
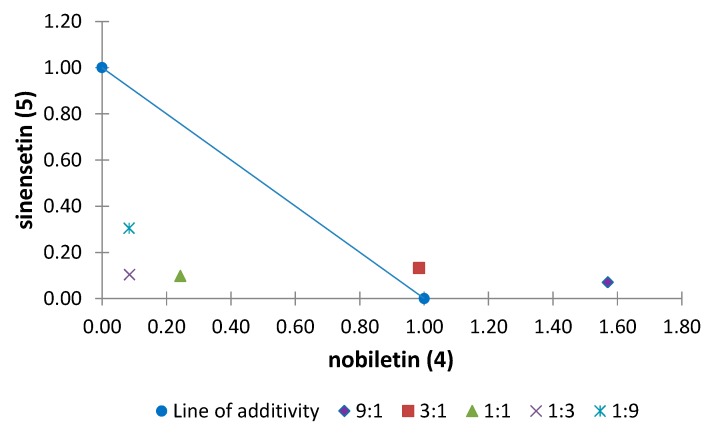
Normalized isobologram (ED_25_) for the binary mixture nobiletin (**4)**/sinensetin (**5**) in the etiolated wheat coleoptile bioassay. (Combination ratios 9:1; 3:1; 1:1; 1:3; 1:9). The combination ratios 9:1 and 3:1 were not significantly different when Student’s *t* test was applied and therefore an additive effect was not evident. The proportions 1:1, 1:3 and 1:9 are located below the line of additivity and a significant difference was observed on applying Student’s *t* test, thus showing a synergistic effect.

**Figure 11:**
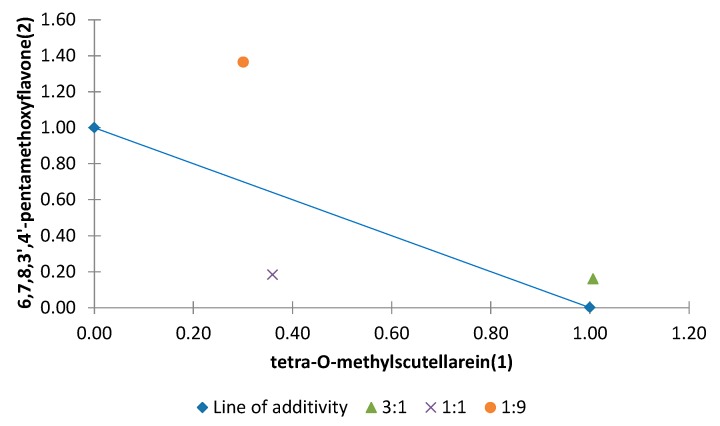
Normalized isobologram (ED_25_) for the binary mixture tetra-*O*-methylscutallarein (**1**)/6,7,8,3′,4′-pentamethoxyflavone (**2**) in the etiolated wheat coleoptile bioassay. (Combination ratios 3:1; 1:1; 1:9). The combination ratio 3:1 did not show a significant difference on applying Student’s *t* test, thus showing an additive effect. The 1:1 ratio is located below the line of additivity and a significant difference was observed on applying Student’s *t* test, thus showing a synergistic effect. The 1:9 ratio is located above the line of additivity and a significant difference was evident on applying Student’s *t* test, thus showing an antagonistic effect.

**Figure 12:**
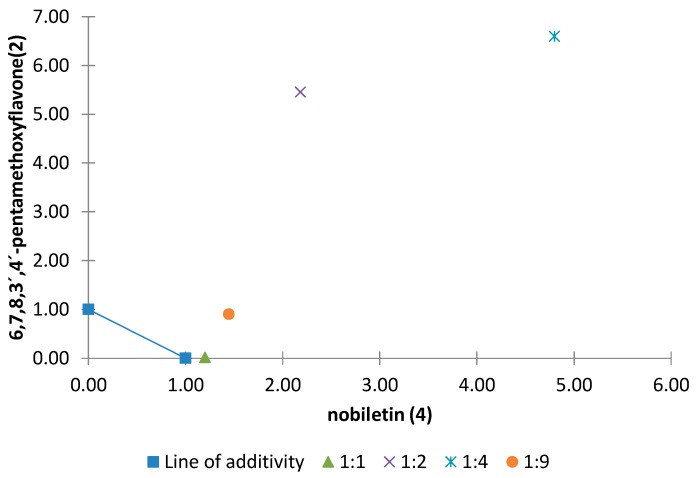
Normalized isobologram (ED_25_) for the binary mixture nobiletine (**4**)/6,7,8,3′,4′-pentamethoxyflavone (**2**) in the etiolated wheat coleoptile bioassay. (Combination ratios 1:1; 1:2; 1:4; 1:9). The ratio of 1:1 did not show any significant difference on applying Student’s *t* test and therefore an additive effect was not found. The 1:2, 1:4 and 1:9 ratios are located above the line of additivity and significant differences were observed on applying Student’s *t* test, thus showing an antagonistic effect.

**Figure 13:**
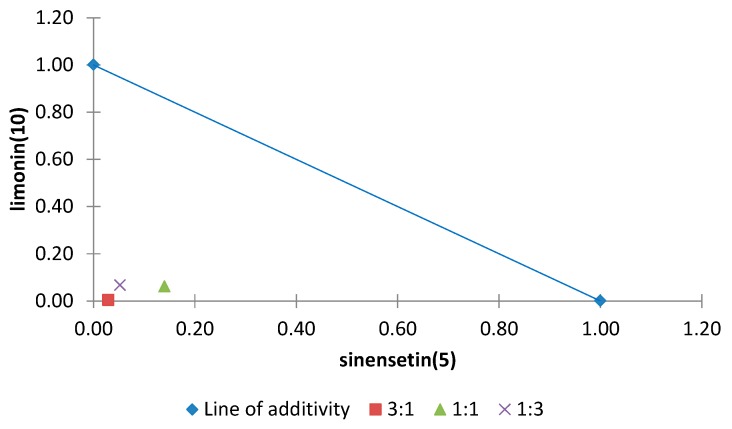
Normalized isobologram (ED_25_) for the binary mixture sinensetin (**5**)/limonin (**10**) in the etiolated wheat coleoptile bioassay. (Combination ratios 3:1; 1:1; 1:3). All ratios are located below the line of additivity and significant differences were observed on applying Student’s *t* test, thus showing a synergistic effect.

**Figure 14:**
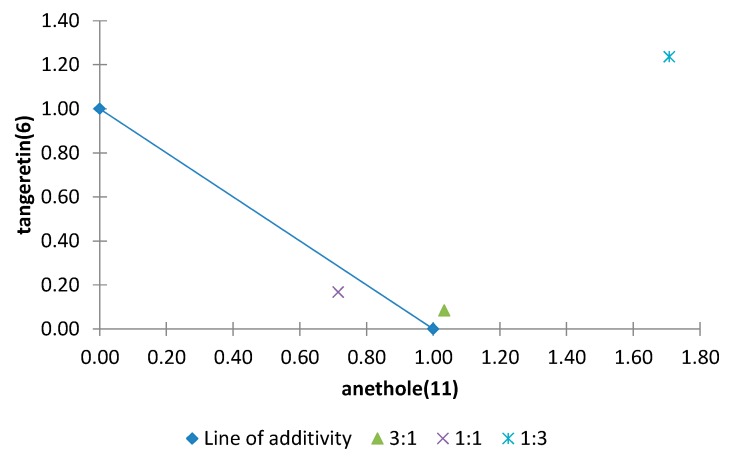
Normalized isobologram (ED_25_) for binary mixtures of anethole (**11**)/tangeretin (**6**) in the etiolated wheat coleoptile bioassay. (Combination ratios 3:1; 1:1; 1:3). The combination ratios 3:1 and 1:1 did not show significant differences on applying Student’s *t* test, thus showing an additive effect. The 1:3 ratio is located above the line of additivity and a significant difference was observed on applying Student’s *t* test, thus showing an antagonistic effect.

Among all the combinations of binary mixtures tested on the elongation of the etiolated wheat coleoptile, the polymethoxyflavones tetra-*O*-methylscutellarein (**1**), nobiletin (**4**) and sinensetin (**5**) showed synergistic effects. Moreover, 6,7,8,3′,4′-pentamethoxyflavone (**2**) and tangeretin (**6**) are involved in the antagonistic effects detected in this general activity bioassay. Normalized isobolograms of binary mixtures for some of these polymethoxyflavones in different proportions are shown in Figure 10, Figure 11 and Figure 12. The effects of interactions between two polymethoxyflavones **5** and **6** with other isolated structures from *Citrus sinensis* were also tested. The normalized isobolograms of binary mixtures of **5** with a limonoid, namely limonin (**10**), and **6** with a phenylpropanoid, namely anethole (**11**), are shown in Figure 13 and Figure 14, respectively.

The results for the binary mixture of nobiletin (**4**)/sinensetin (**5**) show that a synergistic effect is obtained when both compounds are in the same proportion, or **5** is present in a higher proportion. The most notable result was obtained for the combination **4**/**5** (1:3) by virtue of the lowest value for the interaction index (0.2). In contrast, when **4** was present in a higher proportion an additive effect was observed (Figure 10).

The binary mixture that contained tetra-*O*-methylscutallarein (1)/6,7,8,3′,4′-pentamethoxyflavone (**2**) showed different interactions. When both compounds are present in the same proportion a synergistic interaction was detected. However, when **1** was present in a higher proportion there was an additive effect. In contrast, when **2** was present in a higher proportion an antagonistic effect was observed (Figure 11).

The normalized isobologram for the nobiletin (**4**)/6,7,8,3′,4′-pentamethoxyflavone (**2**) binary mixtures in different proportions is shown in Figure 12. The results show that there is an additive effect when the two compounds are present in the same proportion. In contrast, when **2** is present in a higher proportion an antagonistic effect is observed. The most prominent effect was observed for the combination 4/2 (1:4) by virtue of the highest value of the interaction index (λ = 5.3, Table 4).

The normalized isobolograms for binary mixtures between two polymethoxyflavones (**5** and **6**) and two other compounds with different skeletons, also isolated from *Citrus sinensis* (limonoid (**10**) and a phenylpropanoid (**11**)) are shown in Figure 13 and Figure 14. The binary mixture limonin (**10**)/sinensetin (**5**) showed a synergistic effect in all proportions and the most notable effect was observed for the combination **10**/**5** (3:1) by virtue of the lowest interaction index (0.03) (Figure 13). For the binary mixture anethole (**11**)/tangeretin (**6**) an additive effect was found when both compounds were present in the same proportion or **11** was in a higher proportion, whereas when **6** was present in a higher proportion an antagonistic effect was found (Figure 14).

The results obtained with all of the combinations of binary mixtures for these compounds are consistent with the proposal of Caccioni, which considers a holistic approach to explain, for example, the antimicrobial capabilities of citrus essential oils, the activities of which could be the result of a quantitative balance of various components [73] where some of them may act synergistically [74]. Thus, a holistic approach may explain the antimicrobial activities of essential oils, the performance of which may be the result of a quantitative assessment of various compounds where the synergistic and additive effects outweigh the antagonistic effects.

Moreover, a study of the interactions in the elongation of the etiolated wheat coleoptile was carried out in which an active compound (in this case anethole, **11**) was added to a complex mixture (active fractions obtained from dichloromethane and acetone extracts of citrus waste). In this test the activity of the fraction is considered as if it were a single compound. The results obtained are shown in Table 5.

**Table 5:** Types of interactions with Student’s *t* test (α = 0.1) and interaction index (λ) between the different combinations of anethole (**11**) and active fractions (D′′ and E′′) from the acetone extract and (E′–I′) from the DCM extract. (Add.: additive; Syn.: synergistic; Ant.: antagonistic).

Combination Ratio	Mixture of Compounds													
	11:Dʹʹ		11:Eʹʹ		11:Eʹ		11:Fʹ		11:Gʹ		11:Hʹ		11:Iʹ	
	ED50	λ	ED50	λ	ED50	λ	ED50	λ	ED50	λ	ED50	λ	ED50	λ
1:1	Add.	0.8	-	-	Add.	0.8	-	-	-	-	Add.	1.4	-	-
1:2	-	-	Add.	1.4	-	-	-	-	Add.	1	-	-	Add.	1.4
1:3	Syn.	0.4	-	-	Add.	1.4	-	-	-	-	Add.	0.7	-	-
1:4	-	-	-	-	-	-	Add.	1.3	-	-	-	-	-	-
1:6	-	-	-	-	-	-	-	-	Add.	0.9	-	-	Add.	2.1
1:7	-	-	Add.	1.5	-	-	-	-	-	-	-	-	-	-
1:9	Syn.	0.3	-	-	Add.	0.7	-	-	-	-	Add.	0.7	-	-
1:13	-	-	-	-	-	-	Add.	0.8	-	-	-	-	-	-
1:19	-	-	-	-	-	-	-	-	Add.	0.9	-	-	Add.	1.6
1:24	-	-	Ant.	2.5	-	-	-	-	-	-	-	-	-	-
1:32	-	-	-	-	-	-	Add.	0.7	-	-	-	-	-	-

**Figure 15:**
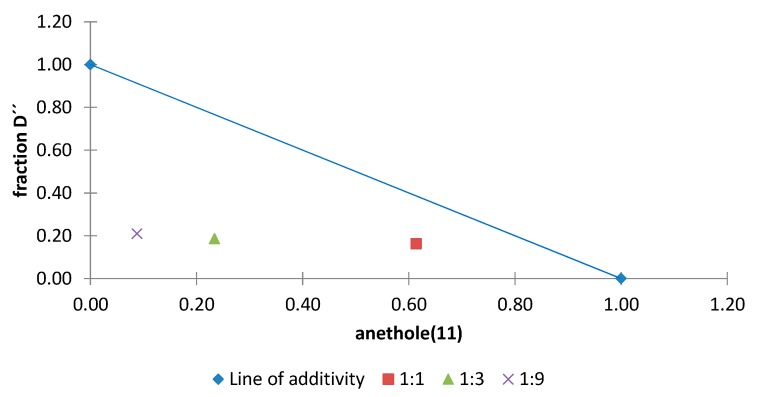
Normalized isobologram (ED_50_) for the complex mixture anethole (**11**)/fraction D′′ (from the acetone extract) in the etiolated wheat coleoptile bioassay. (Combination ratios 1:1; 1:3; 1:9). The mixture with a 1:1 ratio did not show a significant difference on applying Student’s *t* test and this indicates an additive effect. The 1:3 and 1:9 ratios are located below the line of additivity and significant differences were observed on applying Student’s *t* test, thus indicating a synergistic effect.

**Figure 16:**
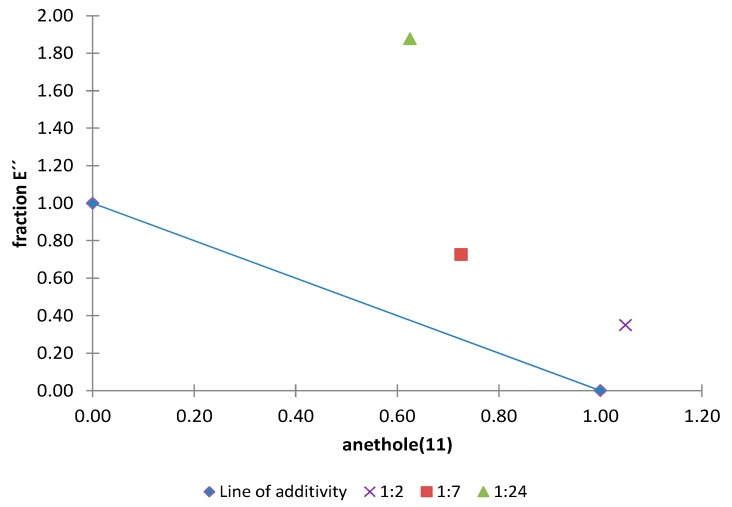
Normalized isobologram (ED_50_) for the complex mixture anethole (**11**)/fraction E′′ (from the acetone extract) in the etiolated wheat coleoptile bioassay. (Combination ratios 1:2; 1:7; 1:24). The 1:2 and 1:7 ratios did not show significant differences on applying Student’s *t* test, thus indicating an additive effect. The 1:24 ratio is located above the line of additivity and a significant difference was observed on applying Student’s *t* test, thus indicating an antagonistic effect.

Of the 21 combinations tested, 19 showed an additive effect, two showed a synergistic effect and one showed an antagonistic effect. Normalized isobolograms for combinations that exhibited synergistic and antagonistic effects are shown in Figure 15 and Figure 16.

The normalized isobologram for the combination of anethole (**11**) with fraction D′′ from the acetone extract in different proportions is shown in Figure 15. The results indicate that a synergistic effect occurs when **11** is present in a lower proportion than fraction D′′ and this is more pronounced for the 1:9 ratio, which has an interaction index of 0.3 (Table 5). Furthermore, the normalized isobologram for the combination of anethole (**11**) with fraction E′′ from the acetone extract is shown in in Figure 16 and an antagonistic effect is observed for the 1:24 ratio.

It is noteworthy that these complex mixtures (with fractions D′′ and E′′), for which synergistic and antagonistic effects were detected, are the same as those that showed strong antioxidant activity in tests with DPPH (Table 3). It can therefore be established that the antioxidant properties due to fractions or compounds present in these mixtures play a role in the interactions between the compounds. Studies have been published in which it is reported that the antioxidant properties of some compounds may act to protect unstable active compounds [23].

On considering the results obtained in this study into interactions, both as binary mixtures or complex mixtures (a total of 65 combinations), it is apparent that most of them showed additive effects (50 combinations) followed by synergistic effects (nine combinations) and, to a lesser extent, antagonism (six combinations). The mixtures investigated were mainly composed of polymethoxy-flavones and, of all the possible interactions, additive and synergistic effects were more prevalent than antagonistic effects, with complex mixtures (with fractions) found to be more active than binary mixtures of pure compounds. These results are also consistent with the proposal by Caccioni concerning the activities of a citrus essential oils extract, which were possibly due to the prevalence of additive and synergistic effects over antagonistic effects [73].

In the analysis of the synergistic and antagonistic interactions detected when anethole (**11**) was added to the active fractions (D′′, E′′) from the acetone extract, the synergistic effect cannot be explained based on the results obtained in the binary combinations performed with 11 and the other compounds present in these fractions (Table 4), since all are effects are additive except for those of the 1:3 anethole (**11**)/tangeretin (**6**) combination, which showed antagonism when **11** was present in a lower proportion than 6, as happens the same in the complex mixture (**11**)/fraction E′′ in a 1:24 ratio (also an antagonistic effect).

This absence of a clear trend based on the binary interactions may be due to the following: (1) the compound in the mixture that produces a synergistic effect with anethole (**11**) has not been identified. (In the case of antagonism in the 1:3 binary mixture (**11**)/(**6**) this could have an influence on the overall effect detected in the complex mixture (**11**)/fraction E′′ in a 1:24 ratio). (2) Interactions with the fractions are not exclusively due to only two compounds and three or more compounds may be involved. In this respect, a more systematic study of the compounds involved in interactions with complex mixtures will be carried out and this will include a fractionation-directed synergistic approach, as proposed by some authors to detect the cause of synergistic effects within complex mixtures [21].

### 2.5. Combination Analysis: Comparing the Additive and Experimental Regression Lines

The isobologram involves the use of sets of equally effective dose combinations for a single effect and it is therefore limited to that specific effect level. In contrast, a more general isobolar analysis that examines combinations of compounds over a range of effects would provide more complete information. Thus, a classification of synergism, antagonism or additivity depends not only on the compound and the effects measured, but also on the fixed ratio combination and the total dose in the combination.

In order to achieve the goal outlined above, a comparison between the theoretical additive curve, constructed from curves of individual compounds for each fixed ratio, and the experimental curve can be performed. The additive regression line and the line obtained from experimental results should be compared in order to assess whether a given interaction type (synergism, additivity or antagonism) is found at some mid-range effect and whether this extends to other dose levels. The F-distribution with a confidence limit of 95% provides a convenient statistic to distinguish whether the two lines differ significantly. The results are shown in Table 6 and Table 7.

**Table 6:** Comparison of the additive and experimental regression lines for binary mixtures of compounds with the F-distribution (α = 0.05) to distinguish two regression lines (if F_cal_ > F there is a significant difference).

Binary Mixture of Compounds	Combination Ratio																	
	9:1		4:1		3:1		2:1		1:1		1:2		1:3		1:4		1:9	
	F_cal_	F (95%)	F_cal_	F (95%)	F_cal_	F (95%)	F_cal_	F (95%)	F_cal_	F (95%)	F_cal_	F (95%)	F_cal_	F (95%)	F_cal_	F (95%)	F_cal_	F (95%)
4:5	0.6	4.82	-	-	1.9	4.82	-	-	74	4.82	-	-	0.1	4.82	-	-	4.0	4.82
4:2	-	-	-	-	-	-	-	-	0.2	4.82	0.1	4.82	-	-	0.1	4.82	0.2	4.82
4:1	3.3	4.82	0.8	4.82	-	-	1.9	4.82	-	-	0.3	4.82	-	-	-	-	-	-
1:2	-	-	-	-	6.6	8.84	-	-	123	6.04	-	-	-	-	-	-	16.9	8.84
1:5	-	-	-	-	1.8	4.82	-	-	0.1	6.04	-	-	-	-	-	-	-	-
5:10	-	-	-	-	3.8	4.82	-	-	3.6	6.04	-	-	5.0	6.16	-	-	-	-
4:10	-	-	-	-	0.1	4.82	-	-	0.3	4.82	-	-	2.8	4.82	-	-	-	-
1:10	-	-	-	-	0.1	6.04	-	-	1.5	4.82	-	-	2.3	4.82	-	-	-	-
11:6	-	-	-	-	0.1	4.82	-	-	0.0	4.82	-	-	0.7	4.82	-	-	-	-
11:1	-	-	-	-	0.1	4.82	-	-	0.1	4.82	-	-	0.1	4.82	-	-	-	-
11:4	-	-	-	-	1.3	4.82	-	-	0.9	4.82	-	-	0.4	4.82	-	-	-	-
11:7	-	-	-	-	0.1	4.82	-	-	0.4	4.82	-	-	-	-	-	-	-	-
11:9	-	-	-	-	0.6	6.04	-	-	0.4	6.04	-	-	0.1	6.04	-	-	-	-
11:16	-	-	-	-	0.1	4.82	-	-	0.0	4.82	-	-	0.5	4.82	-	-	-	-

**Table 7:** Comparison of the additive and experimental regression lines for complex mixtures of compounds with the F-distribution (α = 0.05) to distinguish two regression lines (if F_cal_ > F there is a significant difference).

Combination Ratio	Mixture of Compound													
	11:Dʹʹ		11:Eʹʹ		11:Eʹ		11:Fʹ		11:Gʹ		11:Hʹ		11:Iʹ	
	F_cal_	F (95%)	F_cal_	F (95%)	F_cal_	F (95%)	F_cal_	F (95%)	F_cal_	F (95%)	F_cal_	F (95%)	F_cal_	F (95%)
1:1	0.1	4.82	-	-	1.3	6.04	-	-	-	-	0.0	4.82	-	-
1:2	-	-	0.1	4.82	-	-	-	-	0.1	4.82	-	-	0.1	4.82
1:3	0.1	4.82	-	-	4.4	4.82	-	-	-	-	0.36	4.82	-	-
1:4	-	-	-	-	-	-	0.8	8.84	-	-	-	-	-	-
1:6	-	-	-	-	-	-	-	-	0.1	4.82	-	-	0.1	4.82
1:7	-	-	0.1	4.82	-	-	-	-	-	-	-	-	-	-
1:9	0.1	4.82	-	-	0.9	6.04	-	-	-	-	0.1	4.82	-	-
1:13	-	-	-	-	-	-	0.4	4.82	-	-	-	-	-	-
1:19	-	-	-	-	-	-	-	-	0.0	4.82	-	-	0.8	4.82
1:24	-	-	0.5	4.82	-	-	-	-	-	-	-	-	-	-
1:32	-	-	-	-	-	-	0.4	4.82	-	-	-	-	-	-

**Figure 17:**
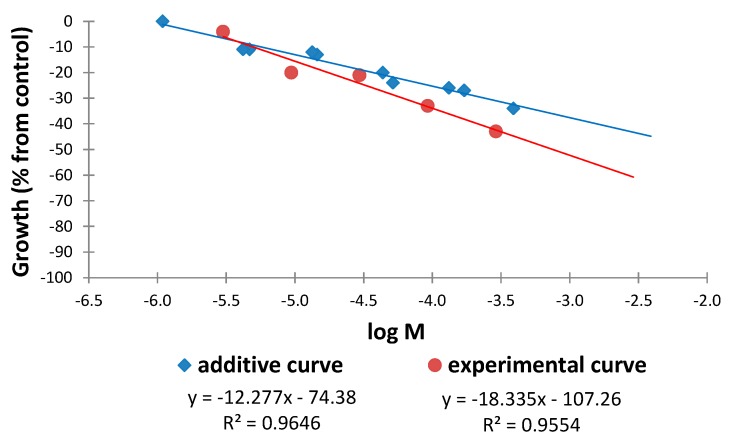
Comparison of the additive and experimental regression lines for the combination nobiletin (**4**)/sinensetin (**5**) in a 1:1 ratio.

**Figure 18:**
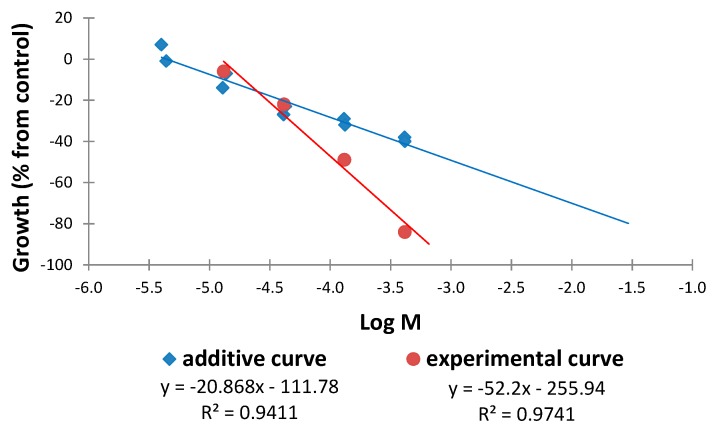
Comparison of the additive and experimental regression lines for the combination tetra-*O*-methylscutellarein (**1**)/6,7,8,3′,4′-pentamethoxyflavone (**2**) in a 1:1 ratio.

**Figure 19:**
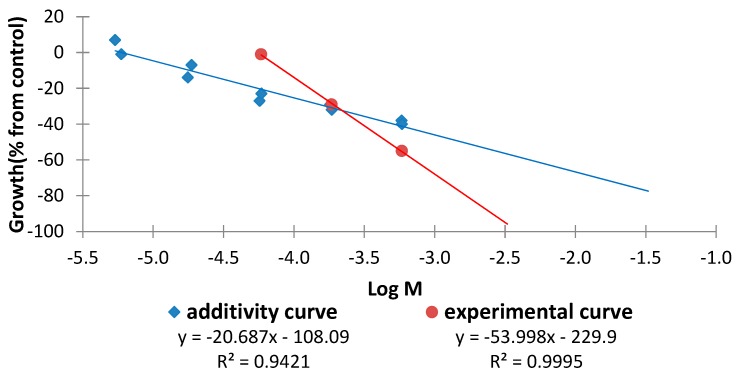
Comparison of the additive and experimental regression lines for the combination tetra-*O*-methylscutellarein (**1**)/6,7,8,3′,4′-pentamethoxyflavone (**2**) in a 1:9 ratio.

For all of the combinations tested, the types of interaction in the isobolograms (ED_50_ or ED_25_) remain constant over different ranges of measured effects. Exceptions to this behavior are the combinations nobiletin (**4**)/sinensetin (**5**) in a 1:1 ratio and the combination tetra-*O*-methylscutellarein (**1**)/6,7,8,3′,4′-pentamethoxyflavone (**2**) in the ratios 1:1 and 1:9, where these interactions were not constant for different ranges of measured effects in the isobolograms (ED_50_ or ED_25_) (Table 4). The additive and experimental regression lines for these binary mixtures in the aforementioned ratios are shown in Figure 17, Figure 18 and Figure 19.

The additive and experimental regression lines for the combination nobiletin (**4**)/sinensetin (**5**) in a 1:1 ratio is shown in Figure 17. It can be observed that with increasing doses of the combination, for the same effect level, the experimental curve shows a smaller dose than the additive curve and that the distance between these lines increases at higher doses. This divergence indicates that a synergistic interaction was detected for this combination in the isobologram (ED_25_) and it is accentuated at higher doses.

Similarly, the results for the combinations tetra-*O*-methylscutellarein (**1**)/6,7,8,3′,4′-pentamethoxyflavone (**2**) in 1:1 and 1:9 ratios are represented in Figure 18 and Figure 19. It can be observed for the 1:1 ratio that increasing doses of the combination, for the same effect level, lead to a smaller dose for the experimental curve than for the additive curve and the distance between the curves increases at higher doses. This finding indicates that a synergistic effect is in operation and at higher doses the difference is more marked. It is noteworthy that for the 1:9 ratio an antagonist interaction is detected in the sobologram (ED_25_) but this changes to a synergistic effect at higher doses. Once again, this interaction is more pronounced at higher doses (Figure 19).

In the three cases where there are differences between the theoretical and the experimental curves, the type of effect observed at higher doses is synergistic and this increases in magnitude with increasing dose. This phenomenon, together with other additive and synergistic effects, is maintained at high doses in different binary combinations of compounds (Table 4) and these are higher than the cases in which antagonism is observed at high doses. These findings may contribute to explain the slight variations observed at higher doses in the activity profiles in the wheat coleoptile bioassays, which highlighted the most bioactive fractions (D, E′, G′, H′, D′′ and E′′) obtained from the different extracts of by-products from *Citrus sinensis* (Figure 3, Figure 4 and Figure 5).

## 3. Experimental Section

### 3.1. General Information

Infrared (IR) spectra (KBr) were recorded on a Fourier transform infrared (FT-IR) Spectrum 1000 spectrophotometer (Perkin-Elmer, Waltham, MA, USA). Nuclear magnetic resonance (NMR) spectra were acquired on 500 MHz and 400 MHz spectrometers (Agilent, Palo Alto, CA, USA). Chemical shifts are given in ppm with respect to residual ^1^H signals of CHCl_3_-*d*_1_ (δ 7.25) and ^13^C signals are referenced to the solvent signal (δ 77.00). HRMS were obtained on a Synapt G2 UPLC-QTOF ESI mass spectrometer (Waters, Milford, MA, USA). Silica gel 0.060–0.200, 60A from Acros Organics (Geel, Belgium) was used for column chromatography. Thin layer chromatography was carried out on TLC silica gel 60 F254 aluminium sheets and TLC silica gel 60 RP-18 F254S aluminium sheets from Merck (Darmstadt, Germany). Compounds were visualized under UV_254/366_ light and by spraying with H_2_SO_4_/H_2_O/HOAc (4:16:80 *v*/*v*/*v*). HPLC was carried out on an HPLC chromatograph (Merck-Hitachi, Tokyo, Japan) with RI detection. Semipreparative HPLC columns (250 mm × 10 mm i.d., 10 µm Lichrospher 250-10 Si60 (Merck) were used in conjunction with a LiChrospher Si60 guard column (Merck).

### 3.2. Chemicals

Hexane, methanol, dichloromethane, ethyl acetate and acetone (Hipersolv Chromanorm for HPLC) were obtained from VWR International (Radnor, PA, USA). MagniSolv Chloroform-D1 (minimum deuteration degree 99.8%) for NMR spectroscopy was obtained from Merck.

### 3.3. Citrus Waste

The material studied was the residue from the production of citrus juice from *Citrus sinensis*, variety Valencia Late, during the campaigns of 2003 and 2004. The frozen citrus residues were supplied by the company LARA (Laranjo Do Algarve-LDA, Sives, Portugal), which is located in southern Portugal. These wastes were sent to CIDEMCO Technology Research Center (Santander, Spain). The samples were thawed over 24 h and then dried in ovens with air circulation for a week at temperatures between 25 and 35 °C. The waste was ground and sieved through a 750 micron mesh and stored at −20 °C. The techniques used to obtain the extracts were steam distillation (to extract essential oils) and maceration of the residues in dichloromethane and acetone (after they were defatted with hexane) in semi-industrial scale installations in EVESA S.A. (La Línea de la Concepción, Cádiz, Spain).

### 3.4. Isolation and Identification of Compounds

The bioactive material under investigation was characterized using a protocol developed in our research group, which involved a wheat coleoptile bioassay in each of the isolation steps (bioassay-guided isolation). The use of this method enables the selection of the extracts and then the fractions that have the best activity profiles and ultimately the isolation, characterization and study of the major secondary metabolites. The three extracts studied from *Citrus sinensis* waste were essential oils, dichloromethane and acetone extracts.

The essential oils extract was bioassayed with etiolated wheat coleoptiles and showed high inhibitory activity and a good activity profile with the dilutions tested. This extract (10 g) was fractionated by column chromatography on silica gel using hexane/ethyl acetate mixtures (0%–100%) as eluent in increasing polarity and ending with 100% methanol. The fractions obtained were concentrated with cooling in an ice bath. The chromatographic separation yielded, after removal of the solvent, four fractions: A (volatile compounds, 8.9 g), B (611.1 mg), C (340 mg) and D (54 mg). Fractions B, C and D showed bioactivity in the wheat coleoptile bioassay and these were purified further by chromatography.

Fractions B and C from the essential oils extract were subjected to column chromatography on silica gel using as eluent hexane/ethyl acetate mixtures of increasing polarity from 0% to 100% and this yielded compound 11 (179.2 mg) as the main component from fraction B and 12 (98 mg) from fraction C. Compounds from fraction D were purified by HPLC using a LiChrospher SiO_2_ (Merck), 10 µm, 250 × 10 mm column, eluting with hexane/acetone in different proportions (hexane/acetone 70:30 and 80:20 *v*/*v*) under isocratic conditions at a flow rate of 3 mL/min. The following amounts of polymethoxyflavones were obtained: **1** (13.9 mg), **3** (1.9 mg), **4** (18 mg), **5** (5.4 mg), **6** (9.5 mg) and **7** (1.8 mg).

The dichloromethane and acetone extracts were bioassayed with etiolated wheat coleoptiles and both showed good bioactivities and inhibition profiles at the dilutions assayed. These extracts were purified by column chromatography on silica gel using as eluent hexane/ethyl acetate of increasing polarity (0%–100%) and by HPLC using a LiChrospher SiO_2_ column (Merck, 10 µm, 250 × 10 mm), eluting with hexane/ethyl acetate in different proportions under isocratic conditions with a flow rate of 3 mL/min. The dichloromethane extract (15 g) provided nine fractions: A′ (1 g), B′ (4.3 g), C′ (697 mg), D′ (462 mg), E′ (507 mg), F′ (805 mg), G′ (1.4 g), H′ (1.3 g) and I′ (1.2 g). The acetone extract (5 g) provided five fractions: A′′ (1.7 g), B′′ (2.1 g), C′′ (93 mg), D′′ (620 mg) and E′′ (448 mg).

Fractions E′, F′, G′, H′ and I′ from the DCM extract showed bioactivity in the coleoptile bioassay and these were further purified by column chromatography. Fraction E′ (507 mg) was subjected to column chromatography on silica gel using as eluent hexane/ethyl acetate mixtures of increasing polarity from 0% to 100% to afford five subfractions: E′1 (25.4 mg), E′2 (81.1 mg), E′3 (84.8 mg), E′4 (78.5 mg) and E′5 (113.2 mg). Subfraction Eʹ2 was purified by HPLC (hexane/ethyl acetate 60:40 *v*/*v*, flow rate 3 mL/min) to yield compound **16** (9.2 mg). Subfraction E′3 was purified by HPLC (hexane/ethyl acetate 60:40 *v*/*v*, flow rate 3 mL/min) to give compound **15** (12.4 mg). Subfraction E′4 was purified by HPLC (hexane/ethyl acetate 60:40 *v*/*v*, flow rate 3 mL/min) to give compound **8** (2.0 mg).

Fraction F′ (805 mg) afforded various subfractions by column chromatography on silica gel using as eluent hexane/ethyl acetate (0%–100%): F′1 (40.2 mg), F′2 (113.7 mg), F′3 (332 mg), F′4 (24.3 mg) and F′5 (42 mg). Subfraction F′2 was purified by HPLC (hexane/ethyl acetate 20:80 *v*/*v*, flow rate 3 mL/min) to give compounds **6** (7.9 mg), **7** (9.6 mg), **9** (2.8 mg), **10** (14.1 mg) and **13** (4.8 mg).

Fraction G′ (1.4 g) afforded six subfractions by column chromatography on silica gel using as eluent hexane/ethyl acetate mixtures of increasing polarity (0%–100%): G′1 (12.7 mg), G′2 (17.2 mg), G′3 (1.2 g), G′4 (44.7 mg), G′5 (30.1 mg) and G′6 (53.7 mg). Subfraction G′2 was purified by HPLC (hexane/ethyl acetate 65:35 *v*/*v*, flow rate 3 mL/min) to give compound **8** (1.4 mg). Subfraction G′3 was purified by HPLC (hexane/ethyl acetate 25:75 *v*/*v*, flow rate 3 mL/min) to yield compounds **1** (52.4 mg), **3** (8.2 mg), **4** (34.6 mg), **6** (33.1 mg), **7** (9.2 mg), **9** (6.4 mg) and **10** (31.6 mg). Subfraction G′4 was purified by HPLC (hexane/ethyl acetate 30:70 *v*/*v*, flow rate 3 mL/min) to yield compounds **1** (2.1 mg), **3** (1.2 mg), **4** (11.7 mg), **7** (1.5 mg) and **10** (8.2 mg). Finally, subfraction G′5 was purified by HPLC (hexane/ethyl acetate 30:70 *v*/*v*, flow rate 3 mL/min) to give compounds **1** (1.6 mg), **3** (1.4 mg), **4** (2.9 mg), **7** (1.6 mg) and **10** (3.2 mg).

Fraction H′ (1.3 g) was subjected to column chromatography on silica gel using as eluent mixtures of hexane/ethyl acetate of increasing polarity from 0% to 100%. A total of seven subfractions were obtained according to their chromatographic similarity: H′1 (5.6 mg), H′2 (26.4 mg), H′3 (254.3 mg), H′4 (509.3 mg), H′5 (123.3 mg) and H′6 (11.6 mg). Subfraction H′2 was purified by HPLC (hexane/ethyl acetate 30:70 *v*/*v*, flow rate 3 mL/min) to give compounds **1** (1.0 mg), **3** (6.0 mg), **4** (1.7 mg), **6** (1.9 mg), **7** (6.5 mg), **9** (4.9 mg) and **10** (1.4 mg). Subfraction H′3 was purified by HPLC (hexane/ethyl acetate 30:70 *v*/*v*, flow rate 3 mL/min) to give compounds **1** (19.7 mg), **3** (16.3 mg), **4** (99.5 mg), **6** (1.7 mg), **7** (4.0 mg) and **10** (1.0 mg). Subfraction H′4 was purified by HPLC (hexane/ethyl acetate 25:75 *v*/*v*, flow rate 3 mL/min) to yield **1** (21.3 mg), **3** (8.2 mg), **4** (201.3 mg) and **5** (102.6 mg). Subfraction H′5 was purified by HPLC (hexane/ethyl acetate 20:80 *v*/*v*, flow rate 3 mL/min) to give **1** (1.6 mg), **3** (1.3 mg), **4** (5.8 mg) and **5** (7.5 mg). Finally, subfraction H′6 was purified by HPLC (hexane/ethyl acetate 20:80 *v*/*v*, flow rate 3 mL/min) to yield **4** (1.6 mg) and **5** (1.5 mg).

Fraction I′ (1.2) afforded six subfractions by column chromatography on silica gel using as eluent hexane/ethyl acetate (0%–100%): I′1 (72.5 mg), I′2 (3.4 mg), I′3 (88 mg), I′4 (251 mg), I′5 (80.3 mg) and H′6 (345.4 mg). Subfraction I′2 was purified by HPLC (hexane/ethyl acetate 50:50 *v*/*v*, flow 3 mL/min) to give **8** (1.2 mg) and **9** (1.4 mg). Subfraction I′3 was purified by HPLC (hexane/ethyl acetate 20:80 *v*/*v*, flow rate 3 mL/min) to yield **1** (2.1 mg), **3** (2.1 mg), **4** (17.2 mg), **5** (20.7 mg) and **6** (1.0 mg). Subfraction I′4 was purified by HPLC (hexane/ethyl acetate 25:85 *v*/*v*, flow 3 mL/min) to give **2** (80.3 mg), **3** (1.8 mg), **4** (3.9 mg) and **5** (9.2 mg). Finally, subfraction I′5 was purified by HPLC (hexane/ethyl acetate 10:90 *v*/*v*, flow rate 3 mL/min) to give **1** (1.0 mg), **3** (1.2 mg), **4** (1.6 mg), **5** (3.5 mg) and **7** (1.4 mg).

Fractions D′′ and E′′ from the acetone extract showed bioactivity in the coleoptile bioassay and they were rechromatographed. Fraction D′′ (620 mg) afforded seven subfractions after column chromatography on silica gel using as eluent hexane/ethyl acetate (0%–100%): D′′1 (12.5 mg), D′′2 (7.7 mg), D′′3 (44.6 mg), D′′4 (38.7 mg), D′′5 (37.9 mg), D′′6 (102.7 mg) and D′′7 (74.1 mg). Subfraction D′′2 was purified by HPLC (hexane/ethyl acetate 80:20 *v*/*v*, flow rate 3 mL/min) to give **14** (5.0 mg). Subfraction D′′4 was purified by HPLC (hexane/ethyl acetate 65:45 *v*/*v*, flow rate 3 mL/min) to yield **9** (1.9 mg). Subfractions D′′5 was purified by HPLC (hexane/ethyl acetate 65:45 *v*/*v*, flow 3 mL/min) to give **8** (1.8 mg). Subfraction D′′6 afforded five fractions after column chromatography on silica gel using as eluent hexane/ethyl acetate (0%–100%): D′′6a (1.8 mg), D′′6b (57.9 mg), D′′6c (19.6 mg), D′′6d (3 mg), D′′6e (5.4 mg). Subfraction D′′6b was purified by HPLC (hexane/ethyl acetate 35:65 *v*/*v*, flow rate 3 mL/min) to give **4** (1.2 mg), **6** (1.6 mg) and **7** (11.8 mg). Fraction D′′6c was purified by HPLC (hexane/ethyl acetate 35:65 *v*/*v*, flow rate 3 mL/min) to give **1** (1.9 mg). Subfraction Dʹʹ6d was purified by HPLC (hexane/ethyl acetate 35:65 *v*/*v*, flow rate 3 mL/min) to give **4** (1.2 mg).

Fraction E′′ (448 mg) was subjected to column chromatography on silica gel using as eluent mixtures of hexane/ethyl acetate of increasing polarity from 0% to 100%. A total of six fractions were obtained according to their chromatographic similarity: E′′1 (22.1 mg), E′′2 (19.4 mg), E′′3 (20.4 mg), E′′4 (49.7 mg), E′′5 (60.6 mg) and E′′6 (70.9 mg). Subfraction Eʹʹ2 was purified by HPLC (hexane/ethyl acetate 30:70 *v*/*v*, flow rate 3 mL/min) to give **6** (1.4 mg) and **7** (2.2 mg). Subfraction E′′3 was purified by HPLC (hexane/ethyl acetate 35:65 *v*/*v*, flow rate 3 mL/min) to give compounds **1** (1.8 mg), **3** (1.2 mg) and **7** (1.4 mg). Subfraction E′′4 was purified by HPLC (hexane/ethyl acetate 30:70 *v*/*v*, flow rate 3 mL/min) to give **1** (3.2 mg), **3** (1.4 mg), **4** (39.4 mg) and **6** (1.2 mg). Subfraction E′′5 was purified by HPLC (hexane/ethyl acetate 20:80 *v*/*v*, flow rate 3 mL/min) to give **1** (1.8 mg), **3** (1.3 mg), **4** (30.7 mg) and **5** (16.6 mg).

### 3.5. Coleoptiles Bioassay

Wheat seeds (*Triticum aestivum* L. cv. Catergo) were sown in 15 cm diameter Petri dishes moistened with water and grown in the dark at 25 ± 1 °C for 3 days [75]. The roots and caryopses were removed from the shoots. The latter were placed in a Van der Weij guillotine and the apical 2 mm were cut off and discarded. The next 4 mm of the coleoptiles were performed under a green safelight [76]. Extracts, fractions and compounds were pre-dissolved in DMSO (0.1%) and diluted in phosphate-citrate buffer containing 2% sucrose at pH 5.6 to the final bioassay concentrations (0.1, 0.5, 0.25, 0.125, and 0.075 mg·mL^−1^ for extracts and 1.0, 0.3, 0.1, 0.03 and 0.01 mM for compounds).

Parallel controls were also run. The commercial herbicide Logran, whose original formulation is a combination of *N*^2^-*tert*-butyl-*N*^4^-ethyl-6-methylthio-1,3,5-triazine-2,4-diamine (terbutryn, 59.4%) and 1-[2-(2-chloroethoxy)phenylsulfonyl]-3-(4-methoxy-6-methyl-1,3,5-triazin-2-yl) urea (triasulfuron, 0.6%), was used as an internal reference at the same concentrations and under the same conditions as reported previously [59]. Buffered aqueous solutions with DMSO and without any test compound were as used as a control for all the samples assayed.

Five coleoptiles and 2 mL of solution were placed in each test tube (three tubes per dilution) and the tubes were rotated at 6 rpm in a roller tube apparatus for 24 h at 25 °C in the dark. The coleoptiles were measured by digitalization of their images. Data were statistically analyzed using Welch’s test [77] and are presented as percentage difference from the control. Positive values represent stimulation and negative values represent inhibition.

### 3.6. DPPH Radical Scavenging Assay

These experiments were performed on a UVmin-1240 UV-Vis spectrophotometer (Shimadzu, Kyoto, Japan) equipped with a multicell (6-cell) and data were processed with UVProbe-Kinetics software.

The procedure is based on “fixed reaction time” of 30 min [78]. A calibration line was constructed with five dilutions (1 × 10^−4^ M; 7 × 10^−5^ M; 6 × 10^−5^ M; 5 × 10^−5^ M; 1 × 10^−5^ M; 1 × 10^−6^ M) with three replicates each. The samples were monitored in a spectrophotometer with a multicell (6 cells) at 515 nm for 10 min, with measurements made at intervals of 2 min. All measurements were performed at room temperature in the dark.

The antioxidant capacity of the samples was measured by adding 3.41 mL of DPPH· 6 × 10^−5^ M to each cell containing the methanolic solution, the blank is measured at 515 nm, followed by 87.4 µL of the sample solution. The cells were then closed and the measurement time was over 30 min at 2 min intervals, with three replicates per sample.

The reduction of the DPPH radical was determined by measuring the absorption at 515 nm. The radical scavenging activity (RSA) was calculated as a percentage of the DPPH discoloration using the equation: % RSA = [1 − (Abst/Abs0)] × 100, where Abs_t_ is the absorbance of the solution when the sample has been added at a particular level and Abs_0_ is the absorbance of the DPPH solution. The extract concentration that provided 50% radical scavenging activity (IC50) was calculated from the graph of RSA percentage against extract concentration. The antioxidant activity index (AAI) was calculated using the equation: AAI = [DPPH·]_0_(µg/mL)/IC_50_(µg/mL), where [DPPH·]_0_ is the initial concentration of DPPH [68]. Gallic acid was used as a standard.

### 3.7. Isobolographic Data Analysis

The dose-effect curves were constructed from data obtained from the coleoptiles bioassay and the curves represent the effect against the logarithm of the dose. The logarithmic transformation resulted in an S-shaped curve that is approximately linear in the mid-range. ANOVA was applied to the linear regression because the slope deviated significantly different from 0 (for log(dose)-effect data this is a test to determine whether the effect is dose-dependent) [79].

An isobolographic analysis was performed to characterize the interaction between the tested compounds and fractions. Isobolographics were constructed using ED_25_ and ED_50_ values (doses that produce 25% or 50% inhibition from the control) obtained when the compound or fraction were administered either alone or in combination. The theoretical additive doses (Z_add_) and the variance (V_add_) for each combination were computed from the equieffective doses (ED_25_ and ED_50_) of the single compounds according to the method described by Tallarida [79]. The experimental data (Z_exp_) and their variance (V_exp_) were determined from the respective dose-response curves. A statistical comparison was made between the experimentally determined (Z_exp_) and the theoretically calculated (Z_add_) values, with Student’s t-test carried out at a 90% confidence limit [79].

The synergism was evaluated from the values of the interaction index (λ), which indicates the proportion of the ED_25_ or ED_50_ value for the individual compound or fraction that accounts for the corresponding ED_25_ or ED_50_ values in the combination, *i.e.*, values close to 1 correspond to an additive interaction, values greater than 1 imply an antagonist interaction, and values less than 1 indicate a synergistic interaction. The interaction index was calculated as follows: λ = Z_exp_/Z_add_ [80].

### 3.8. Data Analysis: Comparing the Additive and Experimental Regression Lines

Data for the dose-effect of the compounds or fractions were used to construct a theoretical additive curve, for each fixed ratio, according to the method described by Tallarida [70]. The linear regressions of the additive and the experimental curves were evaluated by applying a test to distinguish two linear regressions using the F-distribution with a 95% confidence limit [70,78].

## 4. Conclusions

When a reproducible and rapid test system is required, the general wheat coleoptile bioassay is an excellent method to explore not only possible bioactivities of extracts, fractions and products, but also to assess the bioactivities of binary and complex mixtures and to evaluate possible chemical interactions between the compounds. The extraction and isolation of relatively large amounts of by-products from the citrus processing industry provided the bioactive compounds used in this study.

The initial extracts (essential oils, dichloromethane and acetone) showed good activities in the coleoptile bioassay (around 80% inhibition at the highest concentrations) and, as a consequence, a bioassay-guided isolation was performed. This process provided different fractions with good inhibition profiles. The fractions from the acetone extract were the most active in the DPPH radical scavenging assay, with % RSA values between 50% and 80% at the highest concentrations. A total of 16 compounds were isolated from these fractions: nine polymethoxyflavones, two phenylpropaniods, four fatty acids and one limonoid. These compounds were evaluated in the same bioassay and five of them showed better results than their initial fraction and extract, with inhibition values in some cases around 90%, which is similar to that of the commercial herbicide (compounds **1**, **3**, **11**, **12**, **15** and **16**). In a second group of compounds (**4**, **5**, **6**, and **7**) the inhibition levels were around 60% and the majority of these compounds were polymethoxyflavones (**1**, **3**, **4**, **5**, **6**, and **7**). These compounds were tested in the DPPH radical scavenging assay but they did not show antioxidant activity.

In an effort to explain the high bioactivities obtained, the aforementioned extracts and fractions were tested in the coleoptiles bioassay as binary combinations in order to gain a deeper understanding of the interaction effects between compounds in these mixtures. The tests on the binary mixtures were performed on the most bioactive compounds (**1**, **2**, **4**, **5**, **6**, **9**, **10**, **11** and **16**) and with one bioactive compound (**11**) and bioactive fractions (E′, F′, G′, H′, I′, D′′ and E′′). The results obtained in the study of the interactions, both as binary mixtures or complex mixtures (a total of 65 combinations), mostly showed additive effects (50 combinations, 76.9%) followed by synergistic effects (9 combinations, 13.8%) and, to a lesser extent, antagonism (6 combinations, 9.2%). Therefore, of all possible interactions, the additive and synergistic effects were more prevalent than the antagonistic ones and the complex mixtures (with fractions) were more active than the binary mixtures of pure compounds. These results, together with the antioxidant activities, are consistent with the results reported by Caccioni concerning a holistic approach to explain the activities of citrus extracts, which are possibly due to the prevalence of more additive and synergistic effects over the antagonist effects [73].

The results of the study presented here emphasize the importance of experiments on binary mixtures in the coleoptiles bioassay and the potential application, especially for test systems in initial screenings, as a general, rapid and efficient bioassay before studying such binary mixtures by other, more specific bioassays, which can be more laborious and costly. Further study is required to determine the effectiveness of our system and to demonstrate the utility of these bioassays to elucidate the potential effect on many other interactions in binary and complex mixtures.
